# Association of anterior cruciate ligament injury with knee osteoarthritis and total knee replacement: A retrospective cohort study from the Taiwan National Health Insurance Database

**DOI:** 10.1371/journal.pone.0178292

**Published:** 2017-05-30

**Authors:** Sheng-Hsiung Lin, Ting-Chuan Wang, Chun-Fu Lai, Ru-Yin Tsai, Chih-Ping Yang, Chih-Shung Wong

**Affiliations:** 1 Graduate Institute of Medical Science, National Defense Medical Center, Taipei, Taiwan; 2 Department of Medical Research, Cathay General Hospital, Taipei, Taiwan; 3 Division of Occupational Medicine, Department of Family and Community Medicine, Tri-Service General Hospital, National Defense Medical Center, Taipei, Taiwan; 4 Department of Family Medicine, Tri-Service General Hospital Songshan Branch, National Defense Medical Center, Taipei, Taiwan; 5 Department of Nursing, Da-Yeh University, Changhua, Taiwan; 6 Division of Anesthesiology, Armed Forces Taoyuan General Hospital, Taoyuan, Taiwan; 7 Department of Anesthesiology, Cathay General Hospital, Taipei, Taiwan; 8 School of Medicine, Fu-Jen Catholic University, New Taipei, Taiwan; Harvard Medical School/BIDMC, UNITED STATES

## Abstract

**Objective:**

This study aimed to support the potential protective role of anterior cruciate ligament (ACL) reconstruction against the development of osteoarthritis (OA).

**Methods:**

In this retrospective cohort study, the long-term results of ACL reconstruction in Taiwan were evaluated based on data from the National Health Insurance Research Database (NHIRD). In total, 8,769 eligible cases were included from 11,921 ACL-injured patients. The cumulative incidence rates of OA and total knee replacement (TKR) were analyzed using the Kaplan-Meier estimator. Cox proportional hazards models were applied to estimate the hazard ratios (HRs) and 95% confidence intervals (CIs) of OA.

**Results:**

There was a lower cumulative incidence of OA among ACL-reconstructed patients (271, 33.1%) than among non-reconstructed patients (1,874, 40.3%; p < 0.001). Patients who underwent ACL reconstruction had a lower cumulative incidence of TKR during the follow-up period (0.6%) than the non-reconstructed patients (4.6%, p < 0.001). After adjusting for covariates, ACL-injured patients who underwent reconstruction within one month after ACL injury showed a significantly lower risk of OA than those who never underwent reconstruction (adjusted HR = 0.83, 95% CI = 0.69–0.99).

**Conclusions:**

These results indicate that ACL reconstruction might not provide complete protection from OA development after traumatic knee injury but does yield a lower cumulative incidence of OA development and TKR. Moreover, based on the present study, ACL-injured patients should undergo reconstruction as early as possible (within one month) to lower the risk of OA.

## Introduction

Epidemiological studies have found that a history of knee injury is associated with an increased risk of osteoarthritis (OA) [1]. A long-term follow-up study reported that people with a knee injury had at least a 5-fold increased risk of developing knee OA [2]. One of the most common knee injuries is anterior cruciate ligament (ACL) injury, for which reconstruction is frequently performed [3–5]. Previous studies have reported an annual incidence of ACL injury in the general population of 0.8 per 1,000 [6]. Each year, more than 100,000 new cases of ACL injury occur, and approximately 75,000 ACL reconstructions are performed in the United States [7]. Knee OA often develops after ACL injury [8]. Studies have reported rates of radiographic tibiofemoral OA as high as 13% for isolated ACL injuries and from 21% to 48% for subjects with combined ACL and meniscal injuries more than 10 years after the injury [9]. However, few Taiwan-based population references are available.

OA is a major cause of disabilities in people aged 65 years and older; current estimates suggest that 40 million people in the United States have OA, and this number increases annually [10]. Similar trends are occurring in Taiwan. Before 1994, health workers in Taiwan conducted a pretested questionnaire to screen for potential disability in 8998 persons aged 20 years and older, and the prevalence of OA in rural, suburban, and urban areas was 6.3%, 5.8%, and 5.1%, respectively [11]. In another study, 2,801,925 patients in Taiwan with a diagnosis of OA in the National Health Insurance Research Database (NHIRD) during a six-year data collection period were examined, and the prevalence of OA was found to increase from 19.24% in 2001 to 23.05% in 2006 [12]. Most elderly individuals with knee pain have limited mobility. Medical treatments for OA include weight loss, nonsteroidal anti-inflammatory drugs (NSAIDs), corticosteroid injections and activity modification [13]. Severe cases require replacement with artificial joints, which not only increase health care costs but also require further manpower to care for the handicapped. Total knee replacement (TKR) is often used for terminal-stage joint diseases of the knee, such as OA [14]. There is adequate evidence that TKR surgery relieves pain and improves physical function [15]. The number of TKR operations increases yearly in developing countries [16]. In Taiwan, the National Health Insurance Administration (NHIA) reported that 12,088 primary TKR surgeries were performed in 2004, a 5.8% increase from the 11,425 surgeries performed in 2003 [15].

The development of OA following ACL injury results from interactions between many risk factors [17], and the factors affecting OA development after ACL reconstruction remain unclear [18]. Several studies have reported that ACL surgery, sex, older age, higher body mass index (BMI), genetic heritage, poor habits and intra-articular injuries are significant risk factors for the development of knee OA [19]. Additionally, a systematic review found that meniscal injuries and meniscectomy were well-documented risk factors for knee OA development after ACL injury [20]. However, other risk factors for OA, such as age, sex, and genetic heritage, cannot be controlled [21]. The condition of the ACL may be a modifiable risk factor for patients with ACL injuries; thus, reconstructive surgery is necessary to prevent future OA [22]. To better understand the effects of ACL reconstruction on the risk of OA, we assessed covariates including sex, age, obesity, meniscus status (meniscus tear, repair surgery, and arthroscopy) and time between ACL injury and reconstruction as risk factors of OA.

ACL reconstruction restores rotational stability to the knee, but whether it prevents knee joint degeneration remains unknown. According to some studies, radiographic evidence of OA has been shown in more than 50% of ACL-deficient knees 5 to 15 years after ACL injury [23,24]. Moreover, post-traumatic knee OA has been shown to occur one year after ACL reconstruction [25]. In fact, several long-term follow-up studies have found that people with knee injuries may be at risk of developing knee OA less than 1 year after ACL injury [17]. We compiled population-based epidemiological data on patients with ACL injury in Taiwan using the NHIRD. The aim of the present study was to obtain strong evidence to support a protective role of ACL reconstruction against the development of OA.

## Materials and methods

### Data sources

We used the NHIRD, which is derived from the National Health Insurance (NHI) program [26]. The NHIRD contains registration records and original claims data for reimbursements in Taiwan. Large computerized databases are generated from the NHIRD by the NHIA and the Ministry of Health and Welfare and are maintained by the National Health Research Institutes; these databases are provided to scientists for research purposes. The NHI program was implemented in Taiwan on March 1, 1995, and provides a mandatory, single-payer social insurance system. This system covers 99.9% of the population, and 93% of the hospitals and clinics in Taiwan are NHI-contracted [26]. The database comprises extensive inpatient and outpatient data on all insured individuals, including sex, birth date, residential or work location, dates of clinical visits, International Classification of Diseases (Ninth Revision) Clinical Modification (ICD-9-CM) diagnostic codes, details of prescribed medications, amount of expenditures and outcome at hospital discharge [27]. In the present study, we used the Longitudinal Health Insurance Database 2000 (LHID2000); this database is a subset of the NHIRD and consists of a random sample of one million people who were included in the NHIRD reimbursement data from 2000, which covered approximately 23.75 million individuals. According to the National Health Research Institutes, there was no significant difference in the distribution of sex (χ^2^ = 1.74, df = 1, p-value = 0.187) between patients in the data subset used herein and in the original NHIRD. The use of big data provides a means to extend the value of studies on OA risk in ACL injury patients with or without reconstruction.

### Study design and population

**Fig 1:**
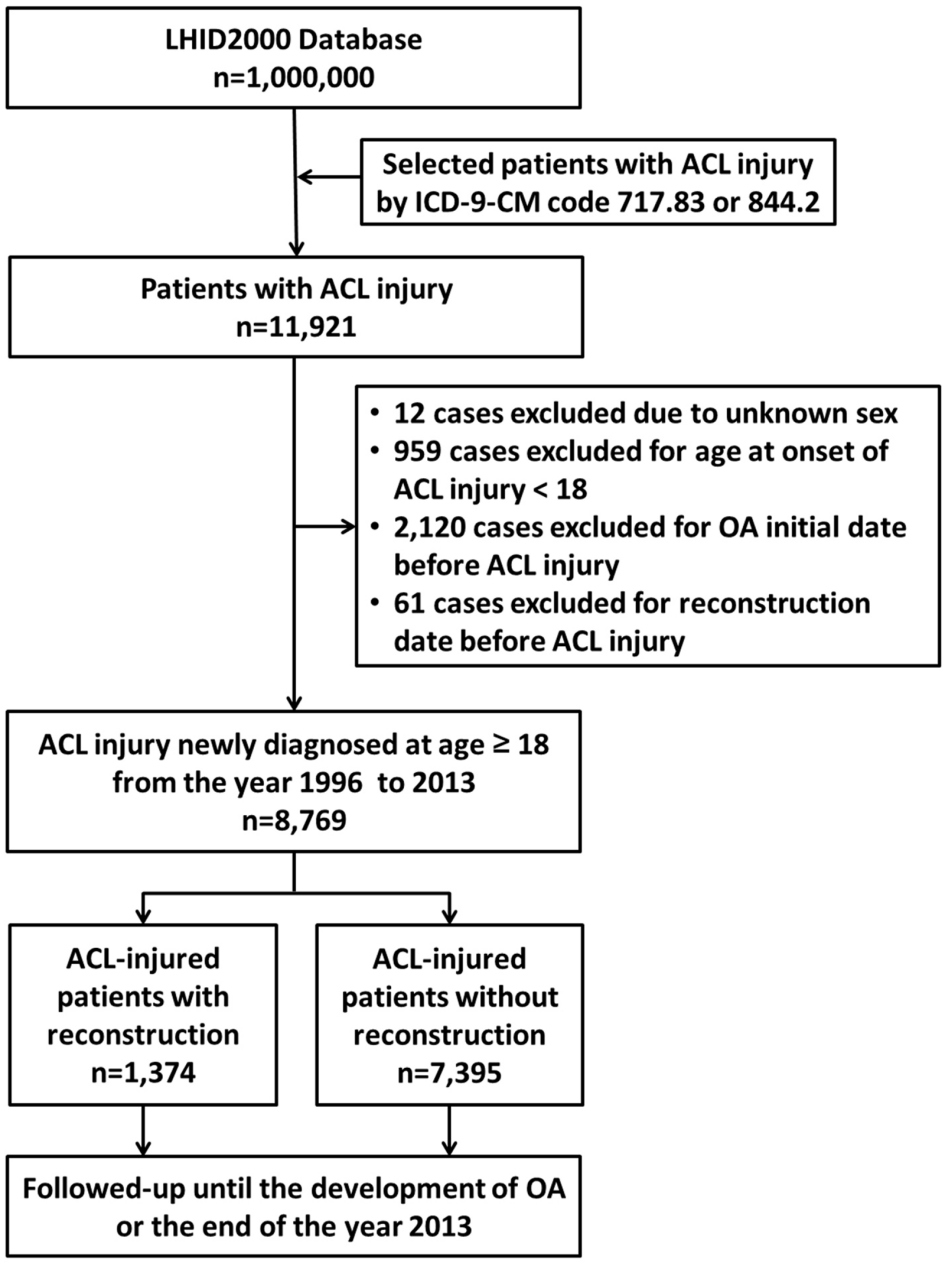
Flow diagram of the study cohort selection and study design.

A retrospective population-based cohort was evaluated in the present study. The study cohort comprised patients with ACL injury who underwent ACL reconstruction surgery and a reference group of patients with ACL injury who did not undergo reconstructive surgery during the evaluation period. We evaluated the effects of ACL reconstruction surgery on OA and TKR risk. All ACL-injured patients were selected from the LHID2000, as mentioned above. Patients with ACL injury (defined by ICD-9-CM code 717.83 or 844.2) before December 31, 2013, were included in the analyses. There were 11,921 patients with ACL injury in the database. The first date of diagnosis of ACL injury or OA was defined as the patient’s date of onset. All subjects were followed up from the onset of ACL injury until the onset of OA (defined as ICD-9-CM codes 715.16, 715.26, 715.36, or 715.96) or the end of 2013. We also evaluated the effect of ACL reconstruction on the development of OA in subjects with cartilage or meniscus injury who received meniscectomy, meniscus repair, or arthroscopy-related surgery. The exclusion criteria were unknown sex (12 cases), age less than 18 years at onset of ACL injury (959 cases), date of OA onset prior to ACL injury (2,120 cases), and reconstruction date before ACL injury (61 cases). A total of 8,769 patients with ACL injury were eligible for study inclusion. A flow diagram of the study cohort selection and study design is provided in Fig 1.

### Statistical analysis

All covariates were considered categorical variables, and age was treated as a continuous variable. The data are presented as the mean ± standard deviation (SD). Continuous variables, such as age, were compared using Student's t test. The cumulative incidence of OA and TKR after 18 years of follow-up was estimated for subjects with and without ACL reconstruction using Kaplan-Meier analysis. The risk factors affecting OA development were estimated using Cox proportional hazards models to calculate the hazard ratios (HRs) and corresponding 95% confidence intervals (CIs). All analyses were performed using SAS software version 9.4 (SAS Institute Inc., Cary, NC, USA) and IBM SPSS Statistics version 21 (International Business Machines Corp., Armonk, NY, USA). A p-value < 0.05 was considered statistically significant. Post hoc power analysis was also conducted for the study using G*Power software version 3.1 (Franz Faul, University of Kiel, Kiel, Germany).

### Ethics

This study was approved by the Institutional Review Board of Cathay General Hospital (IRB No.: CGH-P104036), Taipei, Taiwan. All personal identifiers in the NHIRD files from the NHI program were removed before the data were released for this analysis. Therefore, it was impossible for the authors to identify individual data in the database.

## Results

The flow diagram shows that of the 8,769 ACL-injured patients enrolled between January 1996 and December 2013, 1,374 received reconstruction and 7,395 did not (Fig 1). This study included 4,969 (56.7%) male and 3,800 (43.3%) female patients with ACL injury. In total, 2,145 OA cases were diagnosed among the ACL-injured patients, with an incidence of 39.64 per 1,000 person-years out of a total of 54,116 person-years of follow-up, and 159 patients (7.41% of OA cases) underwent TKR. The demographic characteristics of the ACL-injured patients are listed in Table 1. Considering the sample size of 8,769 ACL-injured patients, post hoc power analyses were conducted using G*Power software, resulting in a power of 100% at an α error probability = 0.05 and an effect size of 0.75 [28].

**Table 1:** Baseline characteristics of ACL-injured patients with and without reconstruction.

Variable	ACL-injured patients		p
	With reconstruction n = 1374 (15.7%)	Without reconstruction n = 7395 (84.3%)	
**Sex, n (%)**			<0.001
Male	982 (71.5)	3987 (53.9)	
Female	392 (28.5)	3408 (46.1)	
**Age at ACL injury, mean (SD)**	30.46 (11.30)	40.30 (16.03)	<0.001
**Age at ACL injury, n (%)**			
< 25 years	560 (40.8)	1606 (21.7)	
25–50 years	701 (51.0)	3650 (49.4)	
≥ 50 years	113 (8.2)	139 (28.9)	<0.001
**Obesity, n (%)**	48 (3.5)	245 (3.3)	0.733
**Meniscus tear, n (%)**			
Never	709 (51.6)	6560 (88.7)	
With surgery^a^	479 (34.9)	259 (3.5)	
Without surgery	186 (13.5)	576 (7.8)	<0.001
**Knee arthroscopy**^b^, **n (%)**	552 (40.2)	898 (10.1)	<0.001
**OA patients**^c^, **n (%)**	271 (19.7)	1874 (25.3)	<0.001
**TKR cases**^d^, **n (%)**	5 (0.4)	154 (2.1)	<0.001

^a^ These surgeries included meniscus repair, meniscectomy, and other ACL-associated surgeries.

^b^ Other arthroscopic surgeries, not including meniscus- or ACL-associated arthroscopic surgery.

^c^ These are osteoarthritis (OA) patients.

^d^ TKR: Total knee replacement.

**Fig 2:**
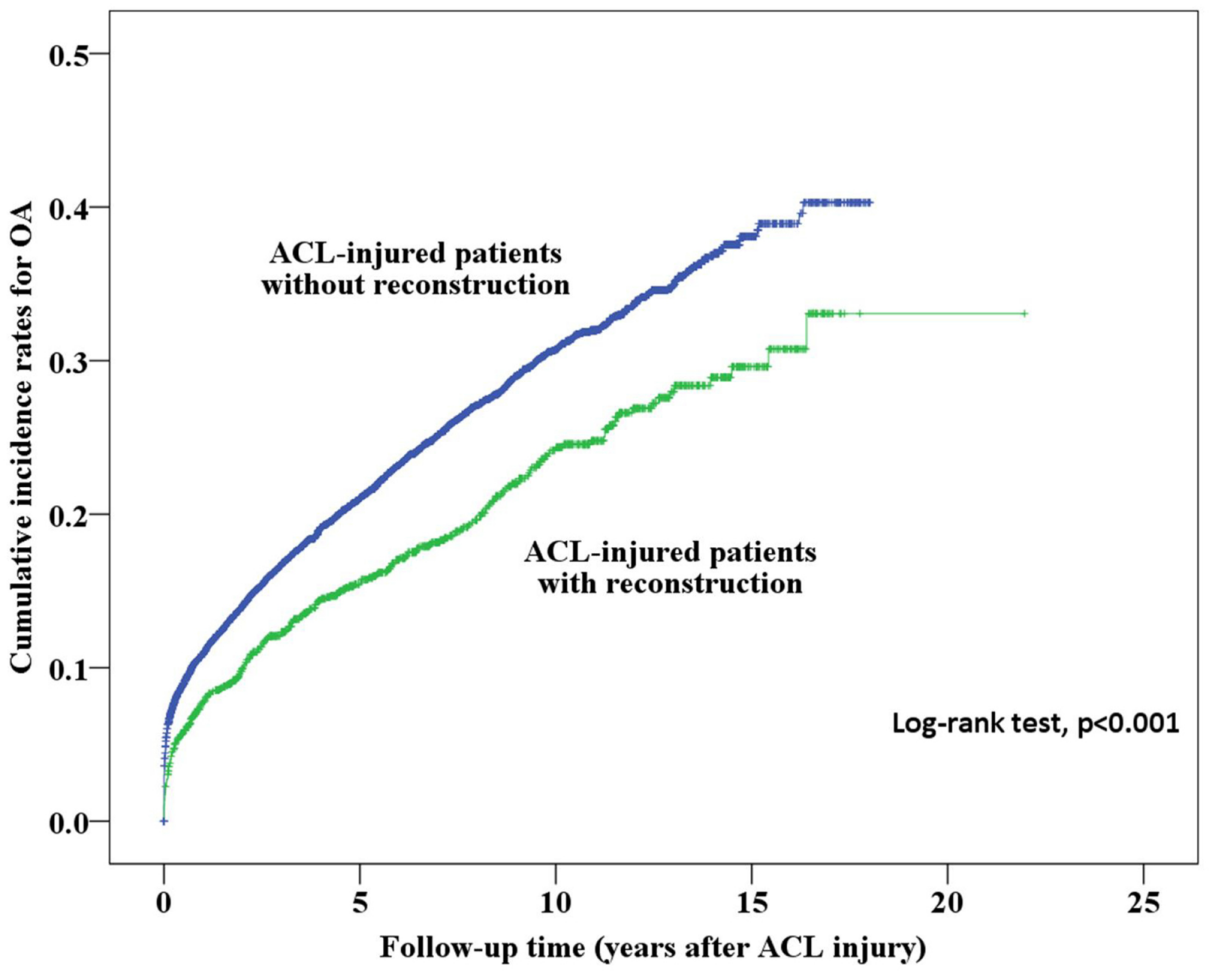
Kaplan-Meier analysis of the cumulative incidence of OA in ACL-injured patients with and without ACL reconstruction surgery.

**Fig 3:**
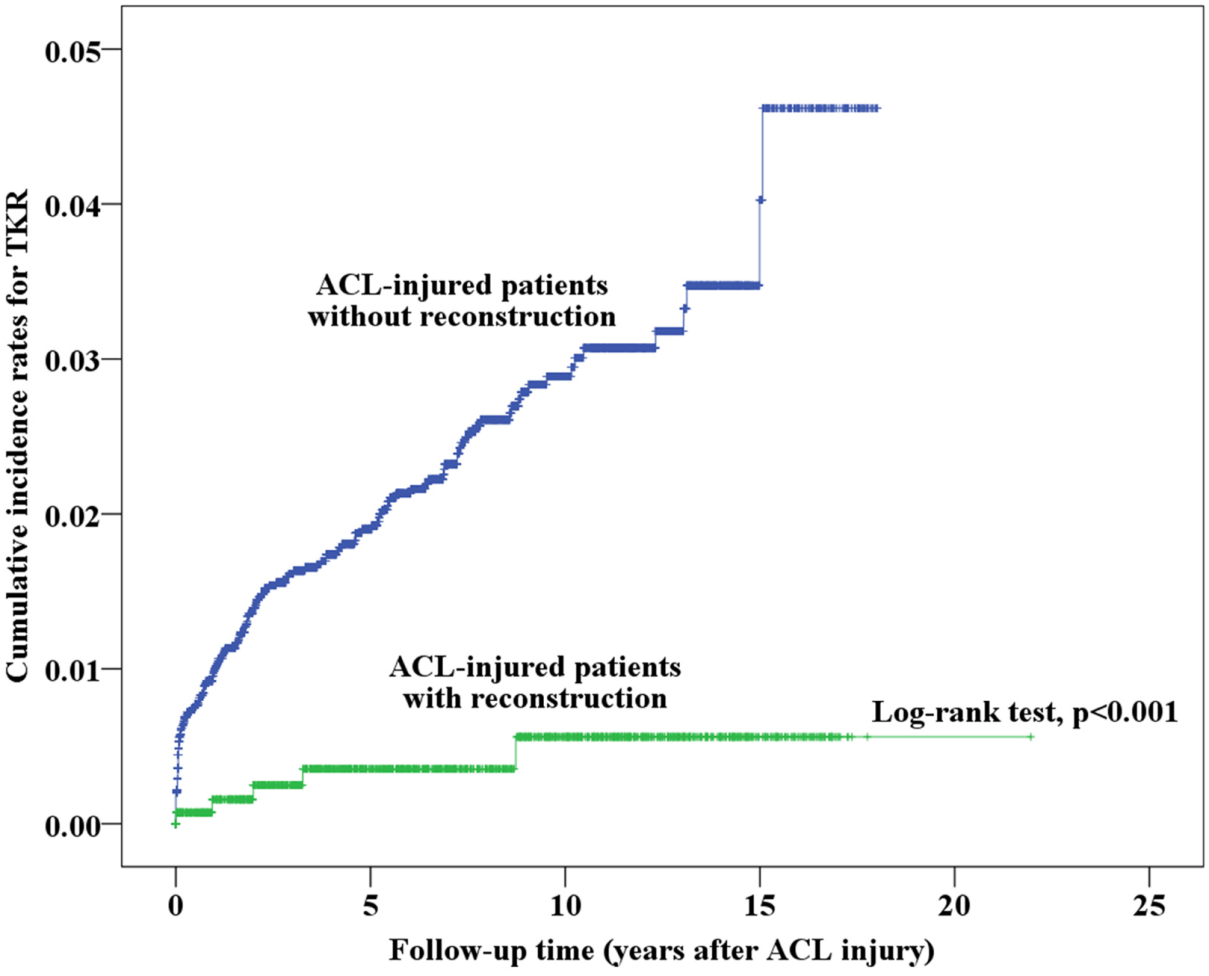
Kaplan-Meier analysis of the cumulative incidence of TKR in ACL-injured patients with and without ACL reconstruction surgery.

Initially, we analyzed the cumulative incidence of OA among patients who did and did not receive ACL reconstruction; the results are shown in Fig 2. The cumulative incidence of OA and the time to onset of OA after ACL injury are presented in Table 2. The mean survival time (years to onset of OA since ACL injury) of patients without ACL reconstruction (13.05 years, 95% CI = 16.24–17.43) was lower than that of patients with reconstruction (16.83 years, 95% CI = 12.86–13.25). ACL patients who did not undergo ACL reconstruction had a much higher cumulative incidence of OA (40.3%) than those who underwent reconstruction (33.1%, p < 0.001). With respect to ACL-injured patients with and without ACL reconstruction, we analyzed 159 patients who underwent TKR of the 8,769 ACL-injured patients. The survival time (years before undergoing TKR after ACL injury) of patients without ACL reconstruction was lower (17.53 years, 95% CI = 17.44–17.62) than that of patients with reconstruction (21.86 years, 95% CI = 21.77–21.95); these results are shown in Table 3. We further computed the cumulative incidence of TKR in patients with and without ACL reconstruction, as shown in Fig 3, and the results showed that reconstructed patients had a significantly lower cumulative incidence of TKR (0.6%) than non-reconstructed patients (4.6%, p < 0.001).

**Table 2:** Cumulative incidence of OA in ACL-injured patients with and without reconstruction.

Reconstruction	Number of patients	Survival time^a^ mean (95% CI)	Cumulative incidence OA events (%)	p^b^
**With**	1,374	16.83 (16.24–17.43)	271 (33.1)	< 0.001
**Without**	7,395	13.05 (12.86–13.25)	1,874 (40.3)	

^a^ Time (years) to onset of OA after ACL injury.

^b^ Log-rank test.

**Table 3:** Cumulative incidence of TKR in ACL-injured patients with and without reconstruction.

Reconstruction	Number of patients	Survival time^a^ mean (95% CI)	Cumulative incidence TKR events (%)	p^b^
**With**	1,374	21.86 (21.77–21.95)	5 (0.6)	< 0.001
**Without**	7,395	17.53 (17.44–17.62)	154 (4.6)	

^a^ Time (years) to onset of TKR after ACL injury.

^b^ Log-rank test.

Moreover, we estimated the HRs and corresponding 95% CIs of sex, age at onset of ACL injury, obesity, meniscus tear or repair, knee arthroscopy, and time to reconstruction after ACL injury in association with OA risk. The results are shown in Table 4. Compared with males, females showed a higher risk of OA, with an adjusted HR of 1.46 (95% CI = 1.33–1.59). With respect to age at onset of ACL injury and OA risk, a significant positive correlation was observed. Compared with patients with an onset of ACL injury at younger than 25 years of age, patients who experienced ACL injury at younger than 50 and at age 50 years and above exhibited an approximately 2-fold (adjusted HR = 2.25, 95% CI = 1.93–2.62) and 7-fold greater risk of OA (adjusted HR = 6.81, 95% CI = 5.84–7.93), respectively. Obesity was associated with a significantly increased risk of OA after ACL injury, with an adjusted HR of 1.30 (95% CI = 1.05–1.61). We then evaluated the effects of cartilage or meniscus injury followed by reparation surgery, such as meniscectomy, meniscus repair, and arthroscopy other than meniscus-related surgery, on OA risk. Compared with ACL-injured patients who had no meniscus tear, the adjusted HR of OA was 1.20 (95% CI = 1.02–1.39) in meniscus-injured patients who did not undergo repair surgery. Finally, we analyzed the association between ACL reconstruction and OA risk. After adjusting for covariates, ACL-injured patients who underwent reconstruction within one month after ACL injury showed a significantly lower risk of OA than those who never received reconstruction (adjusted HR = 0.83, 95% CI = 0.69–0.99).

**Table 4:** HRs for OA development in ACL-injured patients with and without reconstruction.

Variables	No. of subjects	No. of cases^d^	Incidence rate (/1000/year)	HR^e^	
				Crude (95% CI)	Adjusted (95% CI)
**Sex**					
Male	4969	941	29.63	1.00	1.00
Female	3800	1204	53.84	**1.77 (1.63–1.93)**	**1.46 (1.33–1.59)**
**Age at ACL injury**					
< 25 years	2166	214	13.58	1.00	1.00
25–50 years	4351	855	30.87	**2.19 (1.88–2.54)**	**2.25 (1.93–2.62)**
≥ 50 years	2252	1076	100.87	**6.58 (5.68–7.62)**	**6.81 (5.84–7.93)**
**Obesity**					
No	8476	2057	39.25	1.00	1.00
Yes	293	88	51.42	**1.28 (1.04–1.59)**	**1.30 (1.05–1.61)**
**Meniscus tear**					
Never	7269	1746	38.86	1.00	1.00
With surgery^a^	738	199	42.91	1.12 (0.96–1.29)	1.02 (0.85–1.24)
Without surgery	762	200	43.96	1.12 (0.97–1.30)	**1.20 (1.02–1.39)**
**Knee arthroscopy**^b^					
Never	7319	1688	37.52	1.00	1.00
Ever	1450	457	50.04	**1.36 (1.22–1.51)**	**1.78 (1.56–2.03)**
**ACLR**^c^ **time from ACL injury**					
Without ACLR	7395	1874	41.74	1.00	1.00
**< 1 month**	**779**	**135**	25.07	**0.63 (0.53–0.75)**	**0.83 (0.69–0.99)**
1 month– 1 year	410	90	38.97	0.90 (0.73–1.12)	1.21 (0.97–1.52)
1 year– 3 years	103	19	23.38	0.61 (0.39–0.95)	1.07 (0.67–1.69)
≥ 3 years	82	27	38.00	1.02 (0.70–1.49)	1.43 (0.97–2.12)

^a^ The surgery included meniscus repair, meniscectomy, and other ACL-associated surgeries.

^b^ Other arthroscopic surgeries, not including meniscus- or ACL-associated arthroscopic surgery.

^c^ ACLR: Anterior cruciate ligament (ACL) reconstruction.

^d^ These cases involve osteoarthritis (OA) patients.

^e^ HRs: Hazard ratios (95% CI) were calculated using the Cox proportional hazards model.

## Discussion

The present study revealed that the time after ACL injury to OA onset and to TKR were both shorter in patients without ACL reconstruction than in patients with reconstruction. Similarly, the cumulative incidences of both OA and TKR might be reduced when patients undergo ACL reconstruction. Most importantly, the principal finding of this study was that early ACL reconstruction within 1 month of injury might yield a decreased risk of OA development. Furthermore, ACL-injured patients with no history of reconstruction might not have a significantly higher risk of OA development than those who undergo late reconstruction (more than 1 month after ACL injury). Accordingly, ACL reconstruction does not completely protect ACL-injured patients from OA development and severity; instead, we found a decreased cumulative incidence of OA and TKR after ACL reconstruction. We conducted a long-term, 18-year follow-up study of previous ACL injury using the NHIRD to provide additional knowledge and to assist surgeons and patients by facilitating improved decision making.

ACL deficiency enhances OA development [29]. People with acute ACL injury face a risk of OA regardless of whether they undergo early reconstructive surgery. Non-operative therapy and extracapsular augmentation alone or in combination with rehabilitation are also considered treatment options for ACL injury [30]. In our study, 84.3% of the ACL-injured patients in the database did not undergo reconstruction; these individuals might try alternative therapies for an ACL tear, such as rehabilitation prior to surgery. Indeed, ACL reconstruction is well recognized as a strategy to prevent knee joint instability, enabling these patients to return to an active lifestyle [31]. Reconstruction typically restores knee stability and ensures the successful return of function in the short term; however, the long-term consequences vary [32]. We attempted to explore the risks of ACL reconstruction with OA development and adjust for the effects of major risk factors of OA, including sex, age at onset of ACL injury, obesity, meniscus status and knee arthroscopy. Previous studies have demonstrated that patient-related risk factors, such as sex and age, play important roles in the development of OA after ACL injury. Female sex and older age have been positively associated with knee OA risk after ACL reconstruction [24]. Evidence shows that overweight individuals have a high prevalence of knee OA [19,33]. Similarly, in the present study, a correlation between patient-related risks (female and older age) and OA was observed, regardless of whether the patient underwent ACL reconstruction.

Some studies have also illustrated an influence of meniscus damage on the development of OA after ACL injury, showing that approximately 50% of ACL injuries occur in combination with meniscus damage; meniscus tears were treated with arthroscopy in 80% of patients with chronic ACL-deficient knees [23]. A previous study examined the earliest changes that occurred to the ACL in ACL-injured patients by collecting ACL and cartilage samples from autopsies and grading the macroscopic features and histology. The results showed that inflammation, chondroid metaplasia, and cystic changes increased with age [34]. An animal study also showed that middle-aged rats had a higher risk of cartilage degradation after ACL transaction than young rats [35]. These incidents could be biomechanically driven, biochemically mediated or mechanistically influenced through inflammation after joint injury and surgical treatments, including meniscectomy, repair, replacement and even ACL reconstruction [36]. Regarding the association between cartilage damage and OA, meniscal tears and surgical treatment play important roles in the development of OA [36]. A systematic review of 31 studies revealed that the most commonly reported risk factor for OA development was meniscal injury, including meniscectomy, meniscal tear, and meniscal surgery [20]. In the present study, we observed that ACL-injured patients with a history of meniscus tear and surgical treatment had a higher risk of OA. Additionally, ACL-injured patients without surgical treatment of the meniscus after a meniscus tear showed a 1.2-fold increased risk of OA, independent of patient-related risk factors. According to the Cox proportional hazards model, patients with a history of meniscus or joint cartilage injuries might experience OA, regardless of ACL reconstruction.

A retrospective cohort study concluded that operative reconstruction of ACL is better than conservative treatment; surgical reconstruction provided the best strategy for rebuilding normal joint kinematics and structural integrity, thereby reducing the probability of the impaired knee sustaining a secondary injury and degeneration [33]. A study with a follow-up of 17–20 years reported that reconstruction after ACL injury might not completely prevent OA; rather, this strategy reduced the OA prevalence [37]. These results suggested that patients receiving conservative treatment had unstable knees, which increased anterior laxity and led to severe degenerative changes. The results also showed that conservative treatment of additional meniscus injury resulted in a higher prevalence of OA. In contrast, a retrospective cohort study with 11 years of follow-up observed significantly superior knee stability; however, the study also indicated a higher incidence of OA after ACL reconstruction [33]. Indeed, ACL injury alone enhanced the development of OA regardless of whether the patients underwent reconstructive surgery [38]. Thus far, the cause-effect mechanism of the association between ACL injury and reconstruction in OA development remains unknown and depends on risk factors such as sex, BMI, genetic factors, and physical activity [39]. Moreover, the injury severity and reconstructive surgical technique, other than those for meniscus or joint cartilage damage, were also affected in the above study. A previous study reported that compared with those with acute reconstruction, 50% of patients with chronically injured knees showed a significant increase in degenerative changes at the 7-year follow-up [40]. One study reported the occurrence of chronic anterior instability in early knee OA changes, even among patients with a healthy meniscus before ACL reconstruction [40]. That study demonstrated that meniscectomy led to more severe changes. Additionally, the results showed that acute ACL reconstruction with meniscal preservation was associated with the lowest incidence of OA [40]. In the present study, the efficacy of reconstruction was determined after adjusting for meniscal status and patient-related risk factors, and the analyses showed that ACL-injured patients with late reconstruction might not have a higher risk of OA than patients with no history of reconstruction. However, ACL reconstruction was still associated with potential exacerbation of knee joint instability, thereby leading to a decrease in the cumulative incidence of OA in patients undergoing ACL reconstruction. A previous study indicated that early reconstruction of ACL injury may prevent OA in athletes with symptomatic instability [38]. Similarly, we observed a significant decline in OA risk in patients who underwent early ACL reconstruction (within 1 month after ACL injury).

OA is a chronic degenerative disease [13]. The most effective treatment for patients with severe OA is TKR [41,42]. Recent studies have shown that compared to males, females have an HR of 1.58 regarding the risk of knee arthroplasty 15 years after ACL reconstruction [43]. Studies have also reported that older age is associated with an increased risk of OA in post-ACL reconstruction patients still requiring arthroplasty. Patients aged 50 years or older had an HR of 37.28 when compared to patients aged 25 to 29 years with respect to the risk of knee arthroplasty. Accordingly, among ACL-injured patients undergoing ACL reconstruction, being younger and male had a protective effect on the risk of knee arthroplasty [43]. A recent study reported that patients with a history of ACL reconstruction underwent TKR at a younger age (average 50.2 years) than those who underwent other knee surgeries (average 59.9 years), including arthroscopy, meniscectomy, open reduction and internal fixation, chondroplasty, tibial tubercle transfer, and osteotomy [44]. Other studies have reported that patients with prior ACL reconstruction who underwent TKR were younger (average 58.1 years) than patients without ACL injury or prior knee surgery who underwent TKR (average 63.4 years), but this difference was not significant [45]. Similarly, our research showed that the time to TKR after ACL injury was shorter in patients without ACL reconstruction than in patients with reconstruction. The cumulative incidence of TKR was lower when patients underwent ACL reconstruction. As the present study showed that ACL reconstruction also protected patients from OA, it is reasonable to propose that ACL reconstruction might delay the timing of TKR.

The current study has some limitations. First, the NHIRD used ICD-9-CM codes, which do not specify the exact site of injury or surgery, such as the injured side of the knee or OA or the exact knee undergoing reconstruction or TKR. Second, the present study included only patients older than 18 years; therefore, the results are not applicable to younger age groups. Third, the original data from the NHIRD were obtained from a claims-based database. Notably, we could not determine the precise diagnoses of ACL injury, meniscus damage or OA, and these conditions could not be classified according to the degree of severity. Additionally, we were unable to assess how the surgical methods and materials involved in ACL reconstruction and meniscus repair affected OA development. Therefore, TKR was used as the only outcome to indicate the severity of OA. Finally, it was difficult to assess other potential confounding variables, such as BMI and history of knee injury, because the NHIRD lacks information on these variables. Despite these limitations, there are several strengths of the present study: we used a national insurance claims database that covered all medical clinics and hospitals, and a random sample of a consistent percentage of claims was obtained annually from almost all clinics/hospitals in Taiwan. An independent peer group ensured the quality of the diagnoses and medical care through a chart review. Additional strengths of the present study include the large sample size, representativeness, longer follow-up duration, and precise codes for diagnosis and treatment.

## Conclusions

In the present study, we found that ACL reconstruction after ACL injury delayed the time to the development of OA and even TKR. More precisely, ACL reconstruction delayed the incidence of TKR surgery, either by correcting the deformity of the injured knee to prevent the spread of OA throughout the joint or by correcting the overall knee alignment and thus preventing the spread of OA to the contralateral knee. Moreover, we observed that ACL-injured patients without surgical repair after a meniscal tear had a higher risk of OA than those who had a healthy meniscus and underwent surgical repair. ACL-injured patients with a history of knee arthroscopy also had an increased risk of OA. We further observed that patients who underwent reconstruction within 1 month after ACL injury experienced a significant protective effect against OA after adjusting for sex, age at ACL injury, obesity, influence of meniscus damage and arthroscopy. However, ACL with late reconstruction (more than 1 month after injury) did not show evidence of a decreased OA risk. Therefore, ACL reconstruction might not completely protect against OA progression; rather, it might result in a significantly lower cumulative incidence of OA compared with the incidence in non-reconstruction-treated patients. ACL reconstruction was also found to cause a decline in the cumulative incidence of TKR. Based on the present results, we propose further analyzing whether medical treatments or knee injury history, particularly at an early age, affect OA development and subsequent TKR. Such an analysis may provide new strategies for the future management of knee conditions, particularly early management of knee injuries. As demonstrated in our previous serial studies using an ACL transaction and partial medial meniscectomy rat model, early medical intervention delays OA development [46–48]. These findings may provide a new strategy for the early management of knee injuries that may reduce the disease burden, lower the incidence of knee arthroplasty and reduce NHI expenses.
